# Evolutionary conservation of *Trichomonas-*mycoplasma symbiosis across the host species barrier

**DOI:** 10.3389/fmicb.2023.1242275

**Published:** 2023-09-20

**Authors:** Nicholas P. Bailey, Yuxin Shao, Shaodua Du, Peter G. Foster, Jennifer Fettweis, Neil Hall, Zheng Wang, Robert P. Hirt

**Affiliations:** ^1^Biosciences Institute, Newcastle University, Newcastle-upon-Tyne, United Kingdom; ^2^Institute of Animal Husbandry and Veterinary Medicine, Beijing Academy of Agriculture and Forestry Sciences, Beijing, China; ^3^Natural History Museum, London, United Kingdom; ^4^Virginia Commonwealth University, Richmond, VA, United States; ^5^Earlham Institute, Norwich, United Kingdom; ^6^School of Biological Sciences, University of East Anglia, Norwich, Norfolk, United Kingdom

**Keywords:** *Trichomonas gallinae*, *Trichomonas vaginalis*, *Candidatus* Malacoplasma/Mycoplasma girerdii, new bacteria species, *Candidatus* Malacoplasma/Mycoplasma trichamica, metagenomics, metatranscriptomics, phylogenetics

## Abstract

**Introduction:**

The protozoan parasite *Trichomonas vaginalis* is the most common cellular sexually transmitted disease in humans, and the closely related species *Trichomonas gallinae* is an avian parasite of ecological and economic importance. Phylogenetic evidence suggests *T. vaginalis* arose during bird to human transmission of a *T. gallinae*-like ancestor. *Trichomonas vaginalis* shares a strong clinical association with the independent sexually transmitted pathogen *Metamycoplasma* (formerly *Mycoplasma*) *hominis*, and the uncultured bacterium “*Candidatus* Malacoplasma (formerly *Mycoplasma*) girerdii,” with the latter association being an order of magnitude stronger. Both bacterial species have been shown to profoundly influence *T. vaginalis* growth, energy production and virulence-associated mechanisms.

**Methods:**

Evidence for a novel *Malacoplasma* sp. was discovered by *in vivo* Illumina metatranscriptomics sequencing of the *T. gallinae*-infected pigeon mouth. We leveraged published 16S rDNA profiling data from digestive tract of 12 healthy and 24 *T. gallinae*-infected pigeons to investigate association between the novel *Malacoplasma* sp. and *T. gallinae*. We utilised Illumina metagenomics sequencing targeted to pigeon oral and crop samples infected with the novel *Malacoplasma* sp. to generate its full-length genome sequence. Sequence similarity network analysis was used to compare annotated proteins from the novel *Malacoplasma* sp. with a range of other related species.

**Results:**

Here we present evidence for a novel *Malacoplasma* species, related to “*Ca.* M. girerdii,” that is strongly associated with *T. gallinae* in the upper digestive tract of domestic pigeons. Analysis of the genome sequence revealed gene features apparently specific to a *Trichomonas*-symbiotic *Malacoplasma* lineage.

**Discussion:**

These data support a model of long-term association between *Trichomonas* and *Malacoplasma* spp. that has been conserved across diversification of the *Trichomonas* lineage and the host species barrier from birds to human.

## 1. Introduction

Members of the genus *Trichomonas* are microaerophilic microbial eukaryotes and obligate symbionts of birds and mammals within the family Trichomonadidae, class Trichomonadea, phylum Parabasalia (Cepicka et al., 2010). *Trichomonas vaginalis* is the most prevalent cellular sexually transmitted pathogen in humans (Rowley et al., 2019), and is of particular importance due to its association with increased risk of HIV transmission, prenatal and postpartum complications and cervical cancer (Hirt and Sherrard, 2015; Menezes et al., 2016). *Trichomonas gallinae* is an avian parasite of the upper digestive tract (including the mouth and crop; Amin et al., 2014), most strongly associated with columbiforms (Peters et al., 2020) but also found in passerines and raptors (Amin et al., 2014). *Trichomonas gallinae* causes the potentially lethal disease canker in Columbiformes (Amin et al., 2014) and has had major negative impacts on the populations of wild birds, with notably high mortality rates among passerines (Swinnerton et al., 2005; Robinson et al., 2010). However, both *T. vaginalis* and *T. gallinae* are asymptomatic in a large proportion of cases (Seña et al., 2007; Sutton et al., 2007; Chi et al., 2013). A diverse range of other Parabasalids are symbionts of a wide variety of animals including birds, mammals and insects, which includes mutalisitic relationships (Noda et al., 2012).

Phylogenetic evidence strongly suggests that *T. gallinae* and *T. vaginalis* share a bird-infecting common ancestor, and that *T. vaginalis* arose as a result of cross-species transmission from birds to humans (Maritz et al., 2014; Peters et al., 2020). *Trichomonas vaginalis* is most closely related to *Trichomonas gypaetinii* (Martínez-Díaz et al., 2015) and *Trichomonas stableri* (Girard et al., 2014) associated with vultures and band-tailed pigeons, respectively, which form a single lineage within a broad diversity of almost entirely bird-infecting *Trichomonas* sp. (Peters et al., 2020).

Bacteria of the class Mollicutes are generally characterised by their small cell size, reduced genome size and coding capacity, lack of cell wall, and dependence on pathogenic or commensal relationships with microbial eukaryotes, plants or animals (Razin, 2006). Two members of this class, *Mycoplasma hominis* and “*Candidatus* Mycoplasma girerdii” (recently reclassified in the genera *Metamycoplasma* and *Malacoplasma*, respectively; Gupta et al., 2018), share a strong *in vivo* association with the human sexually transmitted parasite *T. vaginalis*, reflecting their symbiotic relationships. The relative risk for co-occurrence of “*Ca.* M. girerdii” with *T. vaginalis* is an order of magnitude higher than for than *M. hominis* (Fettweis et al., 2014), indicating a stronger association with the parasite for the former. *Mycoplasma hominis* can colonise the human urogenital tract in the absence of *T. vaginalis*, is considered a pathogen in its own right, and can be grown axenically (Dessì et al., 2019). In contrast, within the urogenital tract (UGT), “*Ca.* M. girerdii” is found almost exclusively in *T. vaginalis*-infected patients (Fettweis et al., 2014), and *in vitro* cultivation is dependent on the presence of *T. vaginalis* (Margarita et al., 2022), consistent with an obligate symbiosis.

*In vitro* experiments have shown an influential interaction between *M. hominis* and *T. vaginalis*. *Mycoplasma hominis* is able to replicate within *T. vaginalis* cells (Dessì et al., 2005) and the presence of *M. hominis* increases parasite growth rate, has a synergistic effect on the pro-inflammatory response of monocytes, and increases haemolysis by *T. vaginalis* (Fiori et al., 2013; Margarita et al., 2016). *Mycoplasma hominis* and “*Ca.* M. girerdii” were also recently shown to enhance pathogenesis-related processes (hemolysis and adhesion to host cells) and associated gene expression via *in vitro T. vaginalis-Metamycoplasma/Malacoplasma* spp. co-culture experiments (Margarita et al., 2022).

There is also evidence that the *T. vaginalis-M. hominis* symbiosis could have an influence on clinical outcome. It has been suggested that treatment of *T. vaginalis* with metronidazole during pregnancy could cause massive release of *M. hominis*, leading to uterine infection and poorer reproductive outcome (Thi Trung Thu et al., 2018). In addition, *M. hominis* (Dessì et al., 2005) and “*Ca.* M. girerdii” (Margarita et al., 2022) can be protected from the bactericide gentamicin within *T. vaginalis* cells.

Similarly to other Mollicutes spp., “*Ca.* M. girerdii” and *M. hominis* possess small genomes and limited coding capacity, respectively ∼619 kb with ∼572 annotated proteins and ~ 695 kb with ~549 annotated proteins (Fettweis et al., 2014; Calcutt and Foecking, 2015). Genomic analysis suggested a dependence by “*Ca.* M. girerdii” on anaerobic glycolysis and serine and alanine catabolism. In contrast, arginine catabolism appears to be the major energy generation pathway in *M. hominis*. The genomes of both “*Ca.* M. girerdii” and *M. hominis* encode a number of putative host adhesion and virulence-associated factors including BspAs, haemolysins, and collagenases, the latter found only in “*Ca.* M. girerdii” (Fettweis et al., 2014).

In this work, we report the discovery of a close relative of “*Ca.* M. girerdii” inhabiting the oral cavity of domestic pigeons, which shows a strong association with *T. gallinae*, and propose an avian origin for the *T. vaginalis*-“*Ca.* M. girerdii” symbiosis found in the human urogenital tract.

## 2. Materials and methods

### 2.1. Metatranscriptomics sample collection

To collect RNA samples for metatranscriptomic analysis, oral swabs were taken from two adult domestic racing pigeons (*Columba livia*), from the Northeast of England. Birds sampled during this study were routinely treated with antibiotics including ronidazole (antitrichomonal) within 3 months previous to sampling, and showed no signs of disease. All birds were allowed to fly outside daily. A FLOQswab (Copan) was moistened with nuclease-free water and rolled around one half of the inside of the bird’s mouth before suspending in 0.7 mL RNAlater (ThermoFisher Scientific). Swabs were transported on dry ice and stored at −80°C until RNA extraction (within 2 months). Each bird was also screened for the presence of *T. gallinae*. An additional sterile cotton-tipped swab was moistened with sterile distilled water and rolled around the opposite side of the bird’s mouth before suspending in a tube with 13 mL TYM medium (Clark and Diamond, 2002) modified with heat-inactivated horse serum instead of bovine serum, pH adjusted to 7.2, supplemented with 87 μM ferric ammonium citrate, 1 unit/ml penicillin and 100 μg/mL each of streptomycin, nystatin and kanamycin. Tubes were sealed in parafilm and incubated at 37°C. An additional swab was moistened in water and exposed to the air for a few seconds before suspending in identical media as a negative control. Cultures were examined daily under a microscope for the presence of motile trophozoites.

### 2.2. Metatranscriptomics sequencing

Material stored in RNAlater (ThermoFisher Scientific) was thawed on ice, diluted with 0.7 mL nuclease-free PBS and pelleted by centrifugation at 6 k × g for 5 min at 4°C. RNA was extracted from the resulting pellet using TRIzol (ThermoFisher Scientific) according to the manufacturer’s instructions. RNase-free glycogen (10 μg) was used as a carrier during the precipitation phase to improve RNA yield. RNA yield was measured using the Qubit RNA high sensitivity kit (ThermoFisher Scientific), and sample purity and integrity was verified by TapeStation (Agilent) electrophoresis using RNA ScreenTape. To process samples for metantranscriptomic sequencing, total RNA was depleted of rRNA using the QIAseq FastSelect 5S/16S/23S kit (beta version, Qiagen), sequencing libraries were generated using the SMART-Seq Stranded for total RNA-seq kit (Takara) and 75 base pair, paired end reads were generated using an Illumina MiSeq v3 platform.

### 2.3. Metagenomics sample collection

Metagenomics sample collection, processing, sequencing, and data quality control were performed in the lab of Professor Zheng Wang (Beijing Academy of Agriculture and Forestry Sciences). Oral samples were collected from domestic farm pigeons from Beijing, China. A sterile cotton-tipped swab was rolled across the inside of the crop before suspension in 0.5 mL 0.9% saline. Three swabs were collected from each bird. Swabs were used for diagnosis of *T. gallinae* infection by wet mount microscopy; smears were examined by light microscopy for motile parasites. To screen samples for the bacteria of interest by PCR, saline samples from *T. gallinae*-infected birds were centrifuged at 18,630 × g for 5 min, resuspended in 50 μL TE (Tris-EDTA) buffer, heated at 100°C for 5 min and stored at −20°C until required. PCR was performed in 25 μL volumes with 1 × Premix Taq^™^ DNA Polymerase (TaKaRa), 400 nM forward and reverse primer and 2 μL Template DNA. Primers targeted the 23S rRNA gene sequence of the target putative *Mycoplasma* sp. (F: 5’-TAGGACCCGACTAACCCAGA-3′, R: 5’-TTCTGCGCCGAAGATTCAAC-3′). Thermal cycling conditions were performed using a SCILOGEX TC1000-G cyler as follows: initial denaturation at 95°C for 7 min, followed by 35 cycles of denaturing at 94°C for 1 min, annealing at 55°C for 1 min and extension at 72°C for 1 min, with a final extension at 72°C for 10 min. Products were analysed by electrophoresis on a 1.5% agarose gel.

### 2.4. Metagenomics sequencing

Metagenomics sequencing was performed on samples positive for the bacteria of interest. DNA was extracted from saline-suspended swabs using the E.Z.N.A.^®^ Soil DNA Kit (Omega Bio-tek) according to the manufacturer’s instructions. DNA concentration was measured using the QuantiFluor dsDNA System (Promega) measuring fluorescence on a TBS-800 fluortimeter. DNA purity and integrity were assessed, respectively, using a NanoDrop2000 spectrophotometer and by AGE using a 1% gel. DNA was fragmented to approximately 400 bp using a Covaris^®^ M220 ultrasonicator (Gene Company Limited) and used to construct a paired end sequencing library using the NEXTflex^TM^ Rapid DNA-Seq kit (Bioo Scientific). Approximately 40 million 150 bp paired end reads were generated using a NovaSeq sequencer (Illumina). Fastp (Chen et al., 2018) was used to trim adapater sequences and low quality reads (any reads shorter than 50 bp, with average Phred quality score lower than 20, and with any undetermined bases).

PCR was used to close gaps in the resulting metagenomic scaffold and confirm their circularity as described above. The primer pair F: 5’-ATGATTTGAATTGCCCTTCCAG-3′ and R: 5’-AGTTGTTTCGCGTGGTTATG-3′ was used to close a single gap in the target scaffold at an annealing temperature of 56°C. The primer pair F: 5’-GGGATATTGTTATTCCGGCTAAGG-3′ and R: 5’-TCCAAGAGAAGTTACACTATGAGG-3′ was used to confirm scaffold circularity at an annealing temperature of 58°C. Products were sent for GENEWIZ^®^ sequencing by Azenta Biotechnology Co. Ltd. Sequencing was performed in both directions.

We re-sequenced the target bacterial genome using a pigeon oral sample collected for metatranscriptomics analysis (derived from a pigeon from Northeast England). Total DNA was isolated from the remaining material from swab by the Chelex method. Material from the swab tip was excised using a scalpel and was rehydrated by incubating at RT in 300 μL sterile distilled water for 30 min. Swab material was pelleted at 16.1 k × g for 3 min, all but approximately 50 μL of the supernatant was removed, and 150 μL 10% Chelex 100 (Bio-Rad) was added. Samples were vortexed for 15 s, centrifuged at 16.1 k x g for 10 s, incubated at 95°C for 20 min, vortexed again for 15 s, and centrifuged at 16.1 k × g for 3 min. The gDNA-containing supernatant was collected and stored at −20°C until required. Whole genome amplification was performed using a REPLI-g kit (Qiagen), before sequencing library preparation using the KAPA hyper kit (Roche). Sequencing was performed on an Illumina NovaSeq 6,000 sequencer.

### 2.5. Metatranscriptomics data analysis

Quality of raw sequencing data was assessed with fastQC (Andrews, 2018) and adapter sequences were clipped using the fastx-toolkit (Hannon, 2010). Read taxonomy was assigned using Kraken2 (Wood et al., 2019) with default parameters and the NCBI non-redundant nucleotide database as a reference. A *de novo* transcriptome assembly was generated with rnaSPAdes v3.11.1 (Bushmanova et al., 2019) under default parameters, using only reads classified as “*Candidatus* Malacoplasma girerdii.” Assembly quality was assessed by mapping “*Candidatus* Malacoplasma girerdii” reads back to the transcriptome using Bowtie2 (Langmead and Salzberg, 2012), reporting up to 20 alignments per read, and summary statistics were generated using Samtools (Li et al., 2009). Contigs used for downstream analysis were visually inspected as part of an alignment with a range of homologous sequences to check for obvious errors.

### 2.6. Analysis of 16S rRNA profiling

Sequence data from 16S rRNA profiling of the pigeon digestive tract was downloaded from the NCBI’s SRA database using accessions SRR9191928-SRR9192035 (Ji et al., 2020). OTUs (operational taxonomic units), clustered at 97% similarity were generated and quantified by read mapping as described by (Ji et al., 2020). The Ribosomal Database Project Classifier (Wang et al., 2007) was used to assign taxonomy to OTUs at the lowest available taxonomic rank with greater than 70% confidence score. BLASTn search (Altschul et al., 1990) was used to identify OTUs similar to query sequences of interest. Assessment of presence or absence of OTUs of interest between samples was defined by requiring at least 0.1% of total reads to be assigned to the OTU. As indicated in the figure legends, differential abundance of OTUs between conditions was assessed using the ANCOM-BC test (Lin and Peddada, 2020) and the Mann–Whitney U test, the latter using abundances normalised as a percentage of the total library size. ANCOM-BC-derived *p*-values were adjusted to correct for the multiple comparison problem using the Bonferroni method. All statistical analyses were performed in R.

### 2.7. Metagenomics data analysis

Pigeon oral metagenome data was utilised to generate the genome sequence of a newly discovered *Mycoplasma* sp. associated with *T. gallinae*. Quality of sequencing data was confirmed using fastQC (Andrews, 2018). Host-derived reads were filtered from the data by alignment to the *C. livia* reference genome (NCBI genome ID: 10719) using Bowtie2 (Langmead and Salzberg, 2012) with default parameters. A *de novo* assembly was generated from the remaining microbial reads using metaSPAdes (Nurk et al., 2017) with default parameters. To assess circularity and coverage of the resulting scaffolds, paired end microbial reads were aligned to the assembly using Bowtie2 (Langmead and Salzberg, 2012) with default parameters. Sambamba (Tarasov et al., 2015) was used to calculate read coverage across scaffolds using a window size of 2000 bp. Artemis genome browser (Carver et al., 2012) was used to examine read alignment to the assembly and assess scaffold circularity. The Seqinr R package (Charif and Lobry, 2007) was used to calculate GC and tetranucleotide frequency across scaffolds. BUSCO (Bacterial Universal Single Copy Orthologs; Manni et al., 2021) was used to assess genome completeness using the Mollicutes reference dataset. Whole genome alignment was performed using Mauve (Darling et al., 2004) with default parameters.

### 2.8. Phylogenetic analysis

A taxonomic sample of 16S and 23S rRNA genes sequences of interest was obtained by searching the literature and BLASTn search against the NCBI non-redundant nucleotide database (O’leary et al., 2016). Sequences were aligned using Clustal Omega (Sievers et al., 2011) and trimmed using trimAl with a gap threshold of 0.9 (Capella-Gutiérrez et al., 2009). All alignments were visually inspected in SeaView (Gouy et al., 2010). IQ-TREE v2.2.0 (Minh et al., 2020) was used to generate maximum likelihood phylogenies using automatic model selection based on the best fit between the model and data according to the Akaike information criterion (AIC) and Bayesian information criterion (BIC). The selected model is listed in the figure legend for each phylogeny. Branch confidence was assessed by computing 1,000 standard bootstrap replicates. iTOL (Letunic and Bork, 2019) was used to generate annotated figures.

### 2.9. Phylogenomic analysis

The amino acids of 55 protein coding gene (single copy genes) datasets, with 83 taxa each, were aligned with muscle v3.8.31 and sites with poor alignment were identified with BMGE using default settings (version 1.12) and removed (Supplementary Table 2). Prior the phylogenetic analyses any duplicate sequences were then removed. Phylogenetic analyses of these alignments used IQ-TREE, using ModelFinder to choose the best fitting model, and using 1,000 ultrafast bootstrap pseudoreplicates. Each best fitting model is listed in Supplementary Table 2. Taxa that were removed as duplicates in the previous step were then restored to the trees as multifurcations. Compatibility of the gene trees was assessed before concatenating the alignments for those genes. To check for compatibility, we first made a 95% majority-rule consensus tree. Then for each split in that consensus tree we examined each of the 55 gene trees for incompatible splits. In order to be flagged as incompatible, we required that the split in the gene tree have ultrafast bootstrap support of 95% or more, which flagged 1 of the 55 gene trees as being incompatible (Family #2279at2085, Xylose isomerase-like superfamily; Supplementary Table 2). That gene tree was removed, and a 95% majority-rule consensus tree was made with the remaining 54 gene trees, which identified no incompatible trees. The 54 compatible alignments were concatenated for further analysis, making up an alignment of 13,845 sites (Supplementary Data File 2). Phylogenetic analysis of the multi-gene alignment was performed using IQ-TREE v2.2.0. A guide tree was constructed using the LG4X model (Le et al., 2012) with 1,000 ultrafast bootstrap pseudoreplicates, and the final phylogeny was generated using the LG + C20 + F + G model and the PMSF strategy (Wang et al., 2018) with 1,000 ultrafast bootstrap pseudoreplicates.

### 2.10. Gene family analysis

Network analysis was used to predict homologous gene families among the annotated proteins from the genomes of a range of Mollicutes sp. using EGN (Halary et al., 2013). All vs. all BLASTp was used as a similarity search, and genes were clustered into networks with edges linking gene nodes according to BLAST hits. Thresholds for significant BLAST hits were an E value of less than 10–5, percentage identity of greater than 20% and an alignment length of at least 25 AAs and 20% of the length of the shortest sequence and 90% of both sequences. Genes with no BLAST hits above this threshold were considered to be singletons. Networks were assessed using the igraph R package version 1.2.11 (Csardi and Nepusz, 2006). Figures were generated using Cytoscape version 3.6.1 (Shannon et al., 2003).

## 3. Results

### 3.1. Domestic pigeons harbour a relative of “*ca.* M. girerdii” in the upper digestive tract

A *Malacoplasma* sp. related to the *T. vaginalis* symbiont “*Ca.* M. girerdii” was serendipitously discovered during screening of domestic pigeon oral samples in Northeast England. Two pigeon samples were examined for the presence of *T. gallinae* using the culture-based method, one of which was positive for the parasite. One sample for each condition in which *T. gallinae* was present and absent were subjected to metatranscriptomics analysis by Illumina sequencing. Results and metadata are summarised in Table 1. Classification of reads by k-mer alignment showed that the *T. gallinae*-positive sample contained a substantial number of reads with similarity to “*Ca.* M. girerdii,” whereas similar reads in the *T. gallinae* negative sample were almost absent. Reads assigned as *M. hominis* were also virtually absent from both samples, consistent with the absence of this organism. For this data, library rRNA depletion was unsuccessful due to the use of a beta-version of the QIAseq FastSelect 5S/16S/23S rRNA depletion kit (Qiagen), precluding accurate quantification of relative taxonomic abundances in these samples.

**Table 1:** Pigeon samples analysed by metatranscriptomics in this study.

Pigeon ID	Sex	Age (years)	*Trichomonas gallinae* culture test	*n* reads “*Ca.* M. girerdii”	*n* reads *Mycoplasma hominis*
016	M	6	−	23	22
017	M	4	+	40,023	77

**Figure 1:**
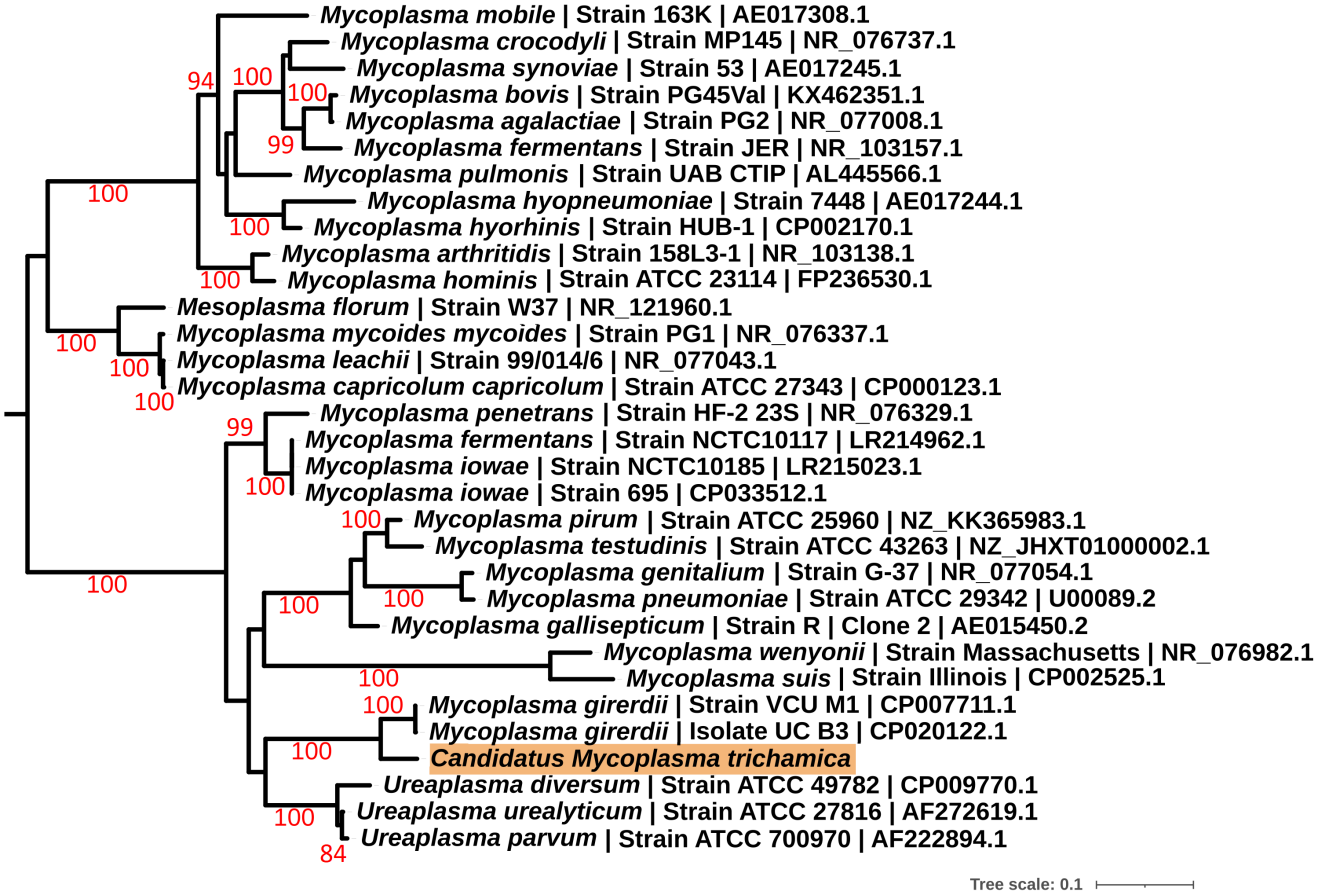
Maximum likelihood phylogeny (GTR + F + I + G) of a 23S rRNA sequence derived from a single pigeon oral metatranscriptome (highlighted in orange) alongside other Mollicutes. Bootstrap support values (1,000 replicates) greater than 75% are shown on branches. The tree is rooted using *Acholeplasma laidlawii* as an outgroup (Pettersson et al., 1996; not shown). Units for tree scale are inferred substitutions per bp. Where available, GenBank accessions are shown at the ends of tip labels.

To further investigate the identity of the putative bacterial species, we generated a *de novo* assembly using the “*Ca.* M. girerdii”-like reads. A total of 15 contigs were generated (Supplementary Table 1; Supplementary Data File 1 with the sequences), and a full-length 23S rRNA sequence was identified from the largest contig by alignment with a range of *Mycoplasma* 23S rRNA sequences. Phylogenetic analysis of the 23S rRNA sequence (Figure 1) revealed that the putative bacteria is a distinct but closely related sister taxon to “*Ca.* M. girerdii,” with maximum bootstrap support.

**Figure 2:**
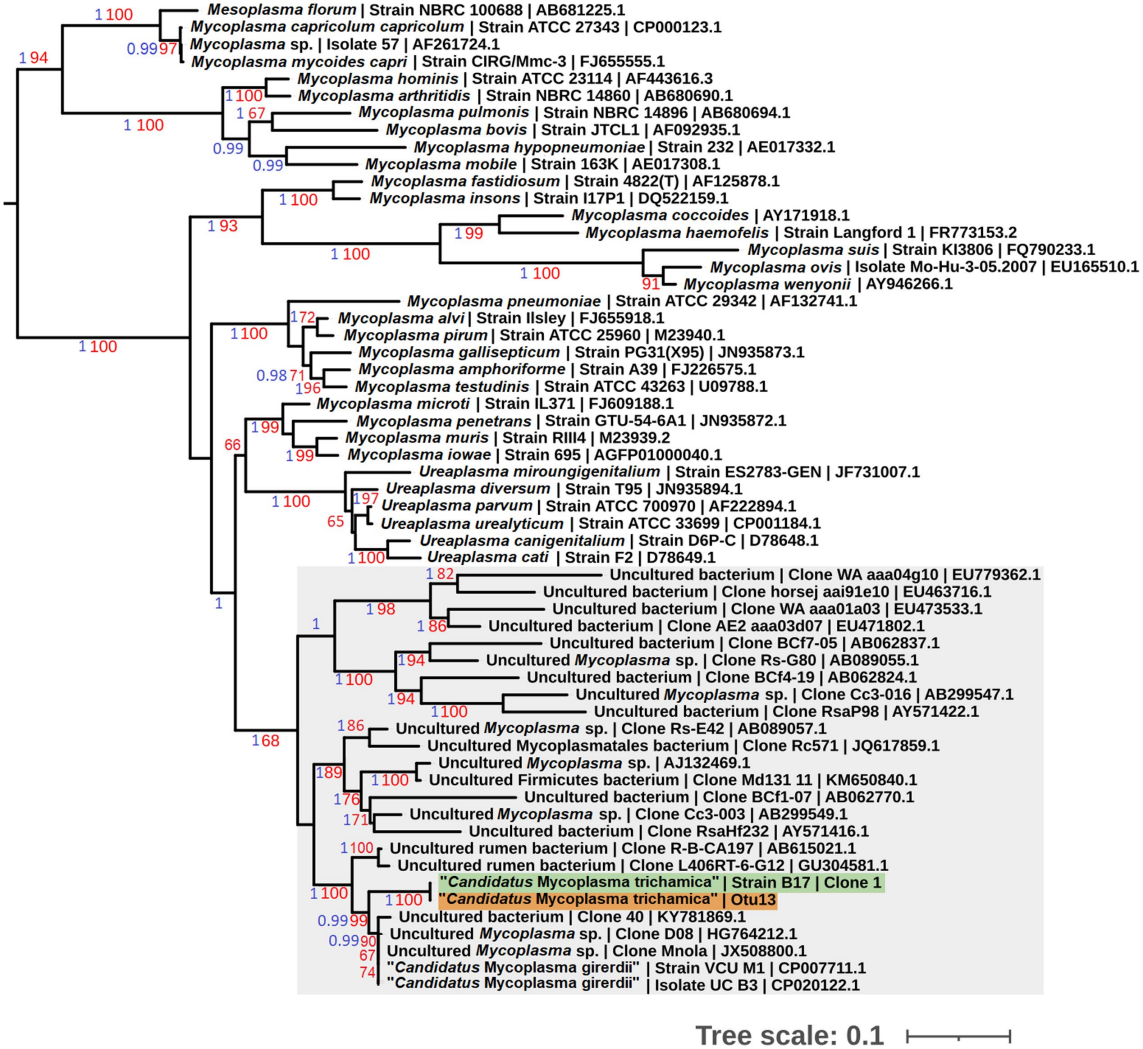
Maximum likelihood phylogeny (GTR + F + I + G) of 16S rRNA sequences derived from bacterial isolates from the pigeon upper gastrointestinal tract (GIT) alongside other Mollicutes. Bootstrap support values (1,000 replicates) greater than 65% and aBayes (Anisimova et al., 2011) values greater than 0.95 are shown on branches in red and blue, respectively. Tree is rooted using *Acholeplasma laidlawii* as an outgroup (Pettersson et al., 1996; not shown). Pigeon-derived sequences from this study and 16S profiling by Ji et al. (2020) are highlighted in green and orange, respectively. Units for tree scale are inferred substitutions per bp. Where available, GenBank accessions for each sequence are shown at the end of tip labels.

To allow sequence comparison with published 16S rRNA sequence profiling we generated a partial 16S rRNA sequence for the bird-associated mycoplasma species. We designed a reverse primer based on the metatranscriptomics 23S rRNA contig used in combination with the generic bacterial 16S rRNA forward primer 338F (Yu et al., 2005; Supplementary Figure 1). We generated a PCR product of the expected size (~1.8 kb), using the metagenomic gDNA template from pigeon 017. The sequences corresponding to the 16S rRNA V3-V4 hypervariable region from 3 independent clones were 100% identical. Phylogenetic analysis demonstrated the pigeon-derived 16S rRNA sequence was closely related to “*Ca.* M. girerdii,” with 99% bootstrap support for the lineage (Figure 2). Herein we describe this putative mycoplasma species as “*Candidatus* Malacoplasma trichamica.”

### 3.2. The putative *Malacoplasma* sp. is strongly associated with *Trichomonas gallinae*

Our preliminary metatransciptomics results were consistent with a putative association between *T. gallinae* and “*Ca.* M. trichamica.” To investigate this further, we searched for “*Ca.* M. trichamica”¬-like 16S sequences among the operational taxonomic units (OTUs) identified in a published 16S ribosomal profiling dataset derived from the crop and gut of 36 farmed pigeons in Beijing, China (Ji et al., 2020). Otu13, showed 100% identity to “*Ca.* M. trichamica” and 97.2% identity to the “*Ca.* M. girerdii” 16S rRNA sequence. In contrast, there were no close hits among the OTUs using *M. hominis* as a query. Consistent with these observations, Otu13 was assigned as *Malacoplasma* (historically called *Mycoplasma*) by the Ribosomal Database Project (RDP) classifier (Wang et al., 2007). A phylogenetic analysis confirmed Otu13 to be closely related to the pigeon-derived mycoplasma 16S rRNA sequence from bird 017 with maximum bootstrap support and “*Ca.* M. girerdii” with 99% support (Figure 2).

Among the cohort of 36 pigeons, 12 each originated from groups heavily infected (high grade; HG), lightly infected (low grade; LG), and uninfected with *T. gallinae* (CG). For each bird, samples were collected from the crop, ilium and rectum (Ji et al., 2020). We assessed the distribution Otu13 among the cohort, to investigate any potential association between *T. gallinae* and the putative *Mycoplasma* sp. (Table 2). Among samples from the crop, Otu13 was found exclusively in birds infected with *T. gallinae*, and was present in the crop of 37.5% of the *T. gallinae*-infected birds. Otu13 was found in only three gut-derived samples, two of which were from birds infected with *T. gallinae*.

**Figure 3:**
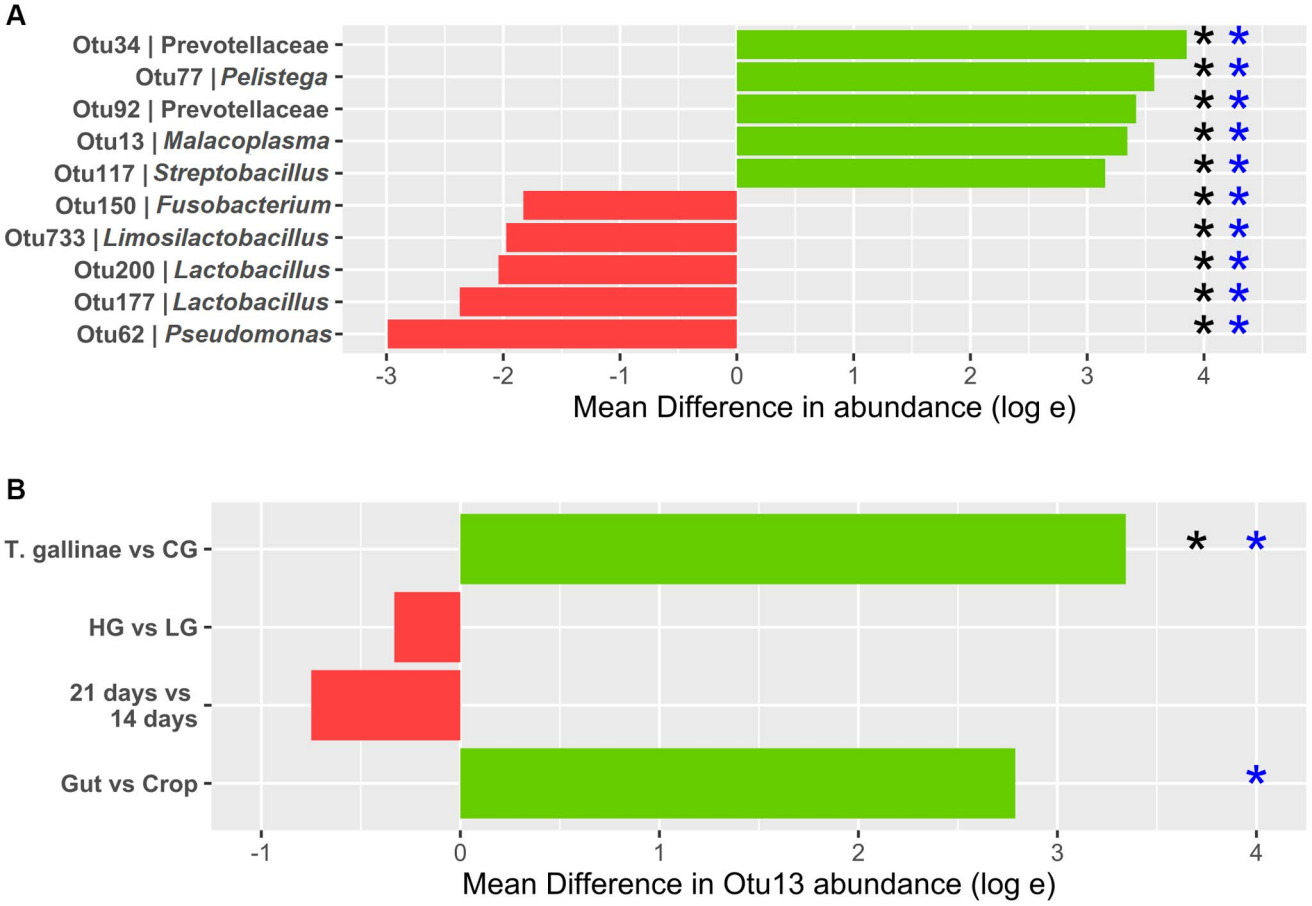
Differential abundance (DA) of OTUs derived from 16S profiling of the pigeon crop **(A)** Top 5 OTUs with the greatest increase and decrease in abundance between the crop of *Trichomonas gallinae*-infected birds (12 total) vs. uninfected controls (24 total). **(B)** Otu13 DA. All comparisons except the gut vs. crop were restricted to crop samples only (12 samples each within the LG and HG conditions, and 18 samples each in the 14-day old and 21-day old conditions). Comparison between the gut and crop samples was restricted to *T. gallinae*-infected birds only (a total of 48 gut-derived samples and 24 crop-derived samples). Significant differences according to the ANCOM-BC and Mann–Whitney U tests are indicated with black and blue asterisks, respectively (value of *p* or *q* value less than 5 × 10^−4^).

**Table 2:** Distribution of Otu13 among a cohort of 36 pigeons sampled in China (data collected by Ji et al., 2020).

Body site	*Trichomonas gallinae* status	*n* Otu13 present
Crop	Negative	0 (12)
	Positive	9 (24)
Gut	Negative	1 (24)
	Positive	2 (48)

Figures in parentheses indicate the total number of samples for each group.

**Figure 4:**
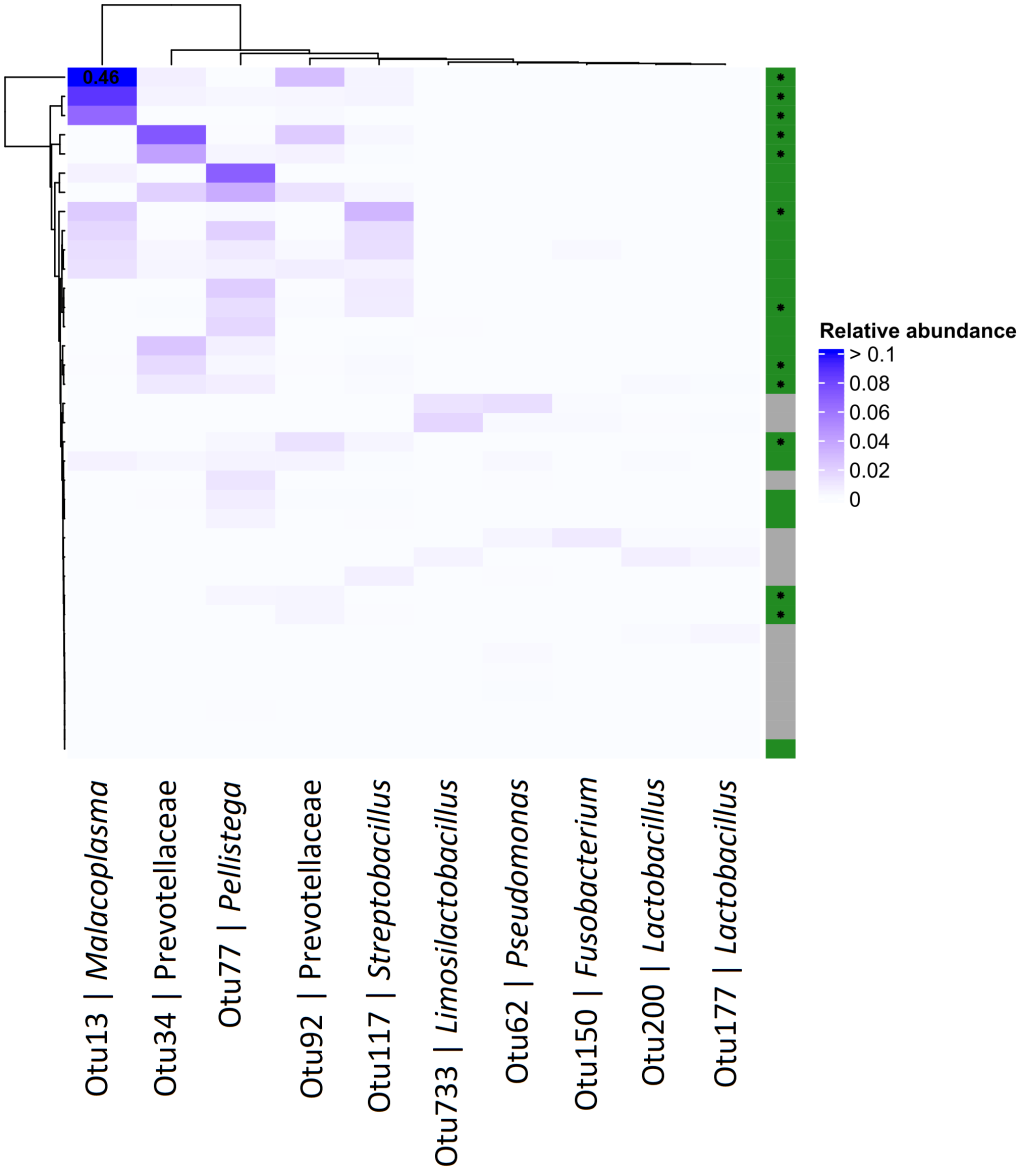
Abundance heatmap for OTUs derived from 16S profiling of the pigeon crop. The top 5 most positively and negatively differentially abundant OTUs in *T. gallinae* infected birds vs. controls, across the crops of pigeon squabs. Green and grey colours on the right indicate *T. gallinae* infected and control birds, respectively, and birds belonging to the HG group are marked with an asterisk. Values greater than 0.1 are indicated in the corresponding cell. Data is derived from 12 samples each from the CG, LG and HG conditions.

Differential abundance (DA) analysis also supported the association (Figure 3). Out of 954 OTUs, 261 were identified to be significantly differentially abundant in the crop of *T. gallinae*-infected vs. control birds (adjusted Q-value less than 0.05). Among all OTUs, Otu13 showed the 4th greatest significant difference in abundance between the crop of *T. gallinae*-infected birds and uninfected controls, although there was no significant difference in abundance between the HG and LG conditions. Interestingly, the abundance of Otu13 was relatively high in several samples (the highest sample abundance was 46% of 16SrRNA reads; Figure 4). Otu13 also showed a somewhat mutually exclusive pattern of abundance with some of the other OTUs most strongly associated with *T. gallinae*, particularly Otu34 (Figure 4). We found no significant difference in abundance of Otu13 between squabs of different ages, however there was tentative evidence that Otu13 was more abundant in the crop than the gut in *T. gallinae*-infected birds, consistent with its co-localisation with *T. gallinae* (Figure 2). Intriguingly, among the 5 most significantly increased OTUs in the *T. gallinae*-infected crop, Otu77 (Figure 2) shares 97% sequence similarity with *Pelistega europaea*, a species isolated from pigeons with respiratory disease (Vandamme et al., 1998). In addition, our results suggested a positive correlation between *T. gallinae* presence and two OTUs assigned to the family Prevotellaceae (Out34 and Otu92) and a negative correlation between *T. gallinae* presence and two OTUs assigned to the *Lactobacillus* genus (Otu177 and Otu200).

### 3.3. Genome sequence of “*ca.* M. trichamica”

**Figure 5:**
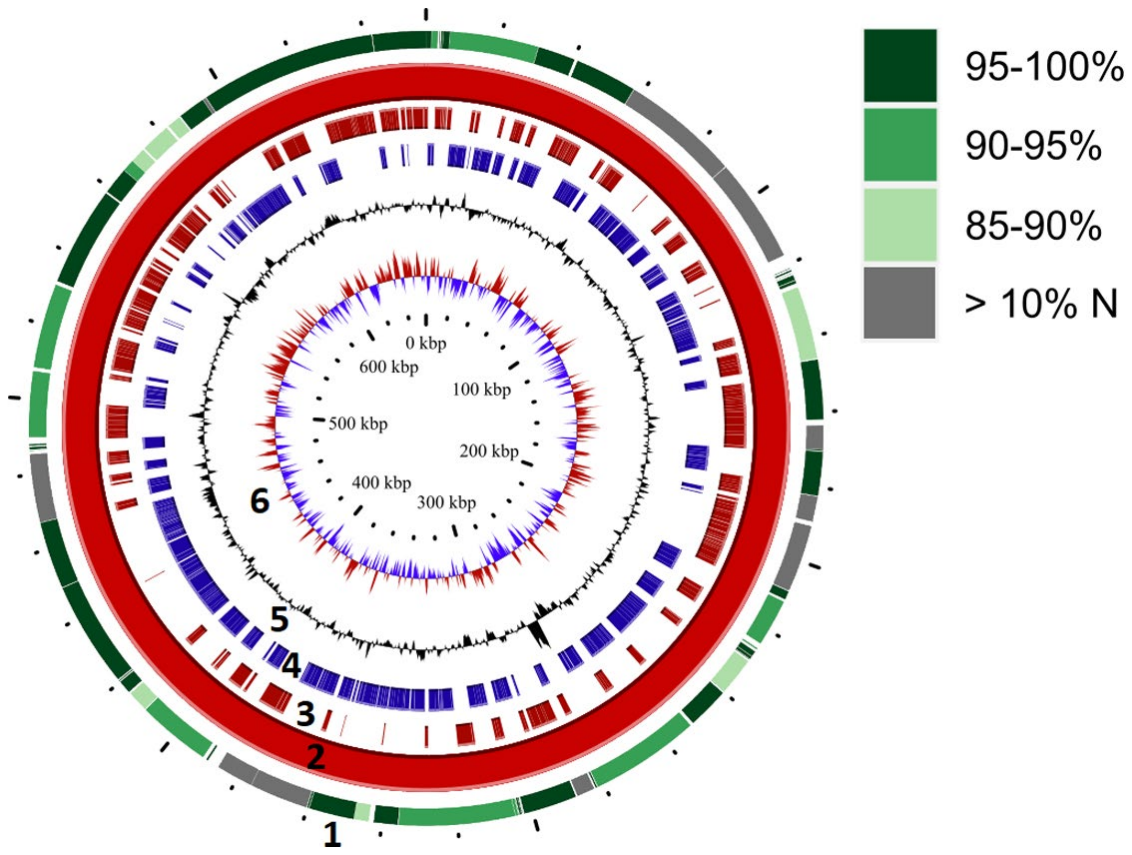
Genome map for “*Ca.* M. trichamica,” derived from pigeon oral metagenomics data. Solid red ring (ring 2) depicts the complete genome sequence derived from Beijing, China. Outer ring (ring 1) indicates segments of the re-sequenced metagenome derived from the UK sample (pigeon 017) which aligned with the complete genome. Green shading indicates the percentage nucleotide identity shared between the two genome sequences, and grey colouring indicates segments for which greater than 10% of bases were undetermined (N). Rings 3 and 4 indicate annotated coding sequences on the plus and minus strands, respectively (ring 2). Rings 5 and 6 indicate GC content and GC skew, respectively (ring 2).

**Figure 6:**
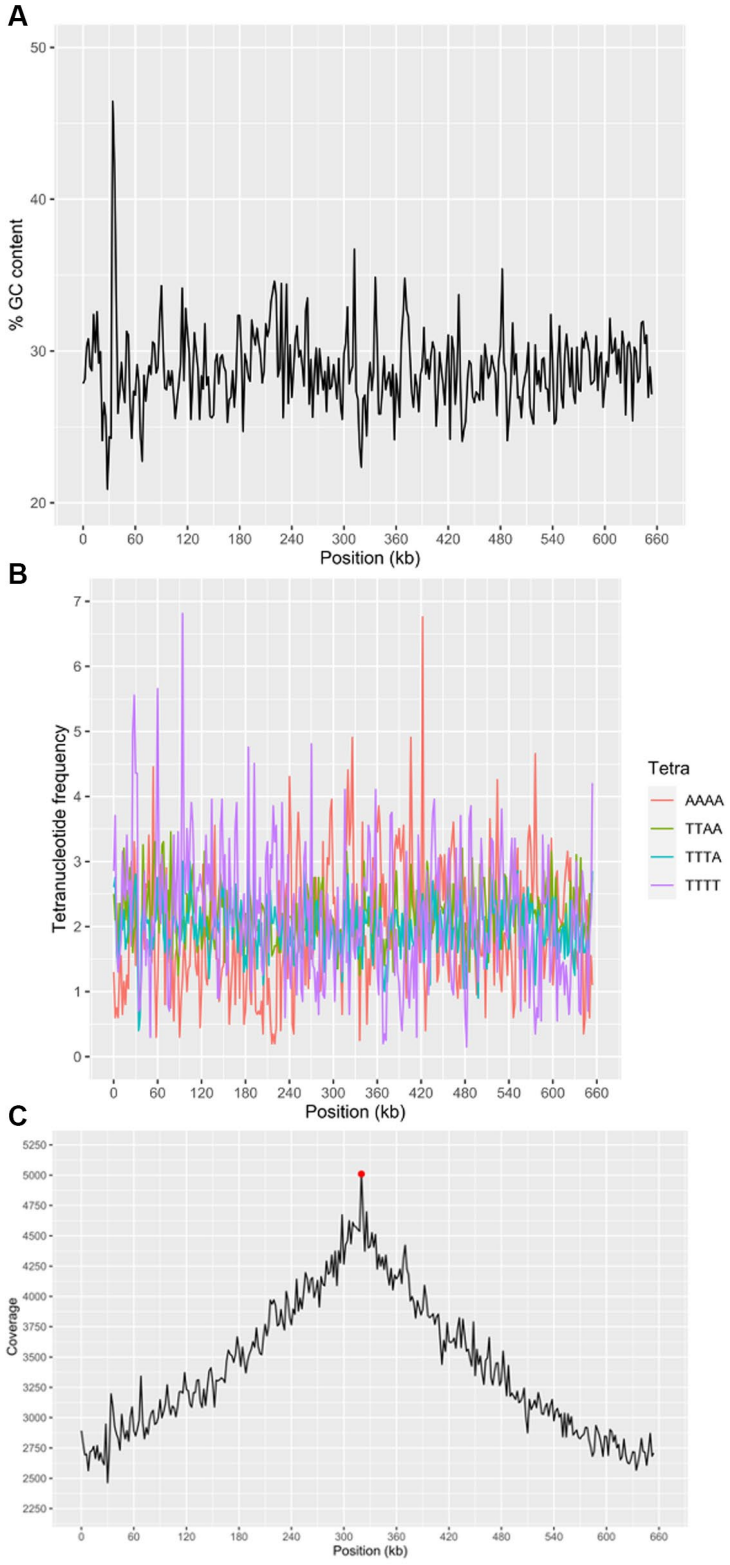
Characteristics of the “*Ca.* M. trichamica” MAG, generated by *de novo* assembly of microbial metagenomics sequences from the crop of a single *T. gallinae*-infected pigeon. **(A)** Percentage GC content across the scaffold. **(B)** Frequency of the 4 most frequent tetranucleotides across the scaffold and **(C)** read coverage across the scaffold assessed by alignment of metagenomic sequence reads to the assembly. Red point indicates the position of the dnaA gene (proximal to the origin of replication). A window size of 2000 bp was used for GC content, tetranucleotide frequency and coverage calculations. “*Ca.* M. trichamica” was artificially linearised to position the putative origin of replication near the centre of the sequence.

We attempted to generate a whole genome sequence for “*Ca.* M. trichamica.” A single domestic pigeon was confirmed to be naturally infected with *T. gallinae* by wet mount microscopy. Infection with the “*Ca.* M. trichamica” was confirmed by PCR using primers designed based on the metatranscriptome-derived ““*Ca.* M. trichamica” 23S rRNA sequence. Metagenome sequencing was performed on a single sample derived from the crop of this bird. After *de novo* assembly of the sequence data, the largest scaffold (scaffold 1) showed characteristics suggesting a near-complete genome. Numerous paired sequence reads spanned between the beginning and end of the scaffold, indicating circularity. A complete metagenomics assembled genome (MAG) sequence was finalised via targeted PCR and Sanger sequencing to confirm scaffold circularity and fill a single ~100 bp sequence gap (Figure 5). The scaffold was ~657 kb, with a GC content of 28.8%, close to the 629 kb, 28.6% GC genome sequence of “*Ca.* M. girerdii” isolate UC_B3 (NCBI assembly accession ASM221542v1). GC content and tetranucleotide frequency were relatively uniform across the MAG (Figures 6A,B), suggesting that the assembly was not chimeric.

We performed a BUSCO (Bacterial Universal Single Copy Orthologs) analysis of the genome completeness, using a reference dataset of Mollicute genes. The results suggested that 147 out of 151 expected single copy genes were present, with an additional putative gene fragment representing one of the remaining 4 genes (annotated as 50S ribosomal protein L15). For comparison, BUSCO analysis of the “*Ca.* M. girerdii” UC_B3 genome revealed 148 out of 151 expected single copy genes were present. The list of expected genes which were entirely absent was identical between scaffold 1 and “*Ca.* M. girerdii.” However, the 50S ribosomal protein L15 existed as a complete gene in “*Ca.* M. girerdii.” We used the genomic location of the dnaA gene as a position indicator for the replication origin, as the two are typically adjacent (Mackiewicz et al., 2004). The dnaA gene was located by tBLASTn (Altschul et al., 1990) search using the DnaA protein sequence from “*Ca.* M. girerdii” UC_B3 (GenBank accession: ASJ88910.1). Coverage across the MAG (proportional to DNA abundance) decreased uniformly from ~5,000X at the putative replication origin to approximately half this value at the opposite region of the presumed circular scaffold (Figure 6C). This suggests that the genome was undergoing replication, indicative of active growth. Consistent with this, 45.4% of non-host metagenomic sequence reads aligned to the MAG, suggesting high abundance of “*Ca.* M. trichamica” in the sampled pigeon.

We re-sequenced an additional isolate of “*Ca.* M. trichamica” via metagenomics derived from the UK sample pigeon 017, from which the initial metatranscriptomics data was generated (Figure 1). Overall, 0.04% of the UK-derived metagenomics reads aligned to the complete MAG, indicating a low overall abundance. A consensus sequence derived from the UK data aligned with the complete MAG with 95.2% coverage and 91.9% overall percentage identity (Figure 5), indicating a high level of similarity between the two genomes as expected.

**Figure 7:**
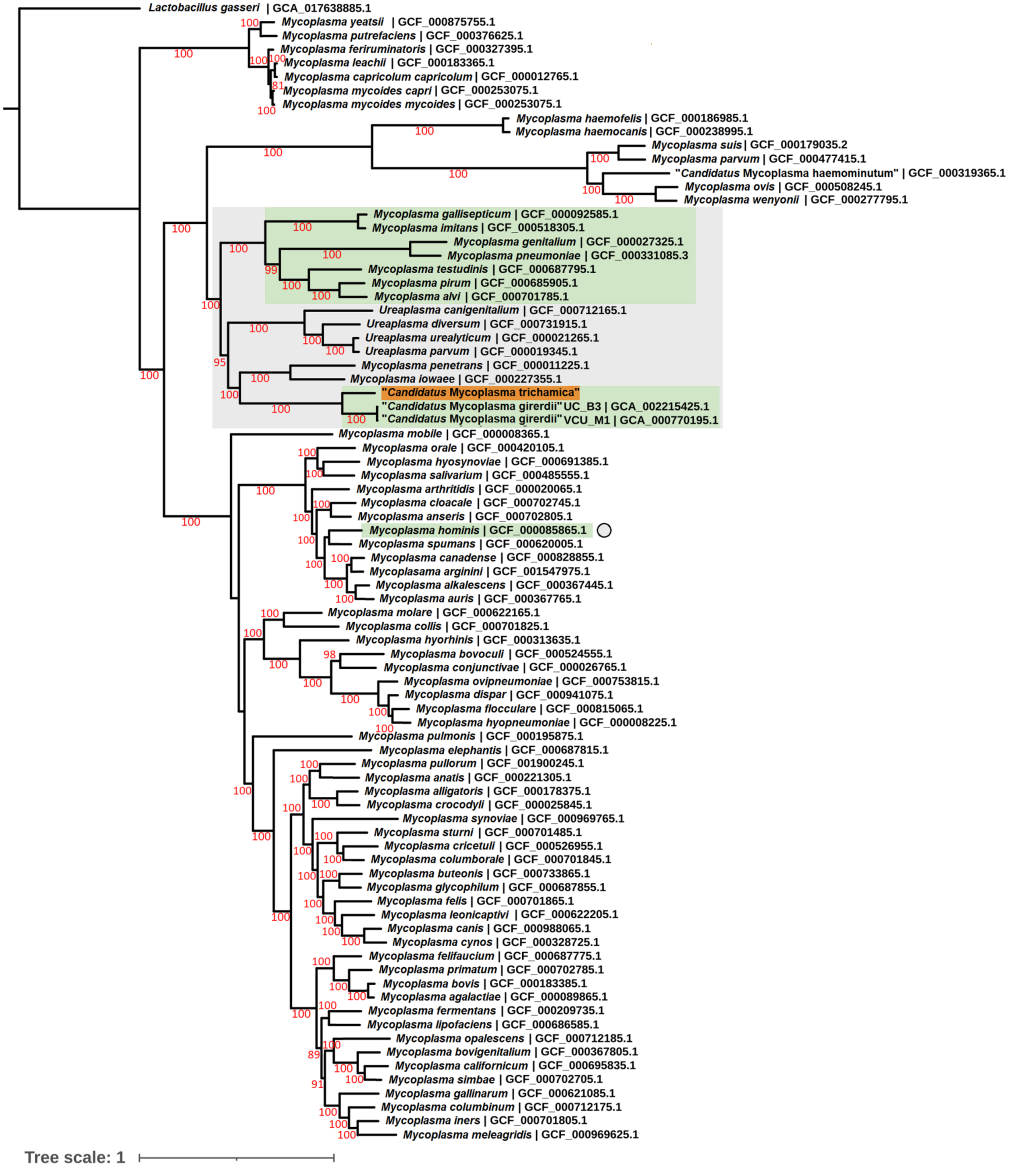
Maximum likelihood phylogeny derived from an alignment of 54 single copy orthologous protein sequences from the “*Ca.* M. trichamica” MAG alongside other Mollicutes. Bootstrap support values (1,000 replicates) greater than 75% are shown on branches in red. Tree is rooted using *Lactobacillus gasseri* as an outgroup (Fettweis et al., 2014). The “*Ca.* M. trichamica” sequence generated in this study is highlighted in orange. The lineages corresponding to genomes encoding putative BspA proteins are highlighted in green. Genome sequences used for gene family analysis are highlighted in grey or marked with a grey circle. Units for tree scale are inferred substitutions per amino acid residue. NCBI genome assembly accessions for each sequence are shown at the end of tip labels.

**Figure 8:**
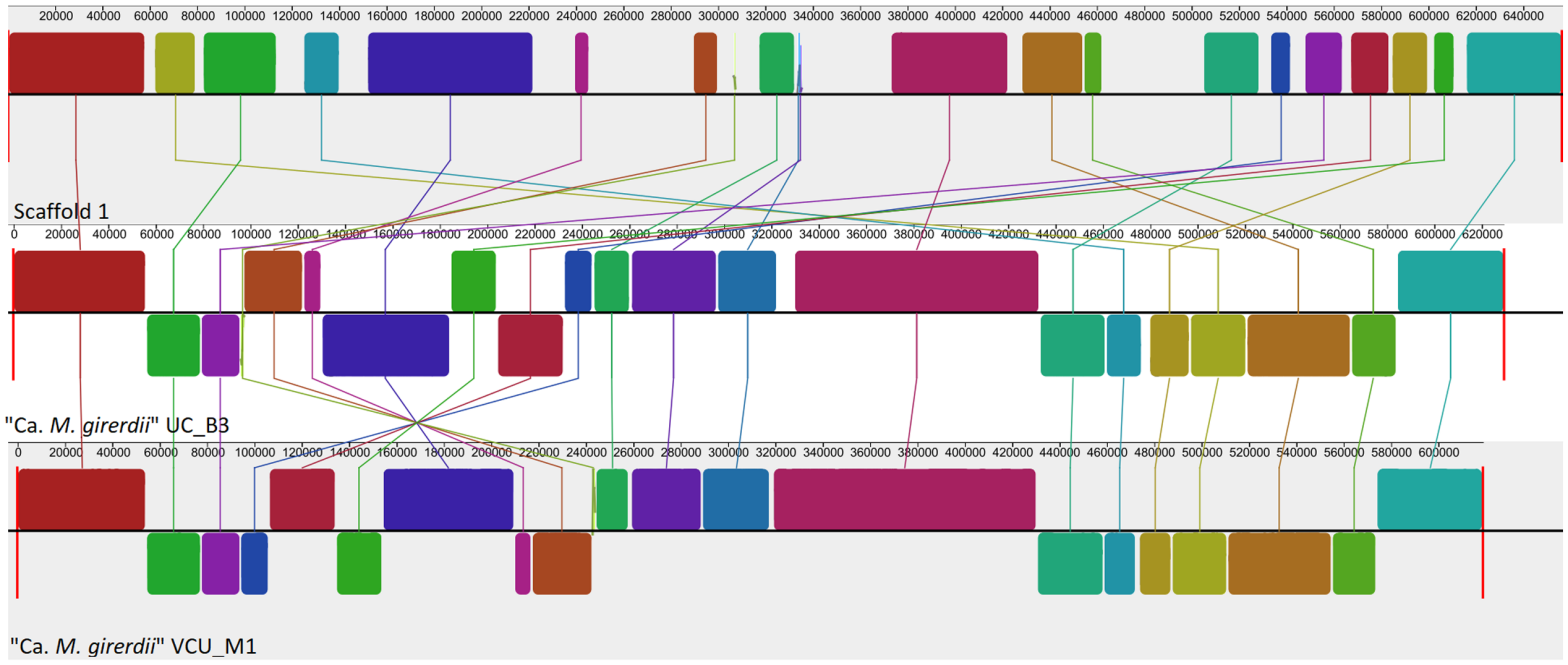
Whole genome alignment of the “*Ca.* M. trichamica” MAG alongside the genomes of “*Ca.* M. girerdii” strains UC_B3 and VCU_M1 (NCBI accessions ASM221542v1 and ASM77019v1), generated using Mauve (Darling et al., 2004). Each coloured block indicates an aligned region between genomes. Blocks above and below the centre aligned in the forward and reverse direction with the “*Ca.* M. trichamica” MAG, respectively.

To complement the rRNA based phylogenies (Figures 1, 3) we performed a phylogenomic analysis exploiting the “*Ca.* M. trichamica” genome sequence using a set of 54 single copy orthologous proteins shared among a diverse sample of available Mollicutes genomes (Figure 7). Consistent with phylogenies based on the rRNA sequence, “*Ca.* M. trichamica” was most closely related to “*Ca.* M. girerdii” with maximum bootstrap support. We performed a whole genome alignment of the “*Ca.* M. trichamica” MAG alongside the two available “*Ca.* M. girerdii” genomes at GenBank (Figure 8). The two “*Ca.* M. girerdii” genomes shared extensive homologous regions and were largely syntenic, although our results supported a large-scale inversion at the ~170 kb position previously described (Costello et al., 2017). The “*Ca.* M. trichamica” MAG shared several large homologous regions with “*Ca.* M. girerdii,” although fewer than between the “*Ca.* M. girerdii” genomes. There appeared to be some syntenic regions between the “*Ca.* M. girerdii” and “*Ca.* M. trichamica” MAGs, but results also suggested extensive genomic rearrangement.

We performed gene family analysis in order to identify potential distinguishing gene features of the “*Ca.* M. girerdii”-like lineage of *Trichomonas*-symbiotic Mollicutes. We included the annotated coding sequences from the “*Ca.* M. trichamica” MAG alongside a lineage of related Mollicutes sequences as defined by the phylogenomic analysis (Figure 8). We also included *M. hominis* due to its relevance as a symbiont of *T. vaginalis* (Dessì et al., 2019). The analysis included 24 Mollicute genomes. Overall, there was a low CDS content; among all genomes analysed, “*Ca.* M. trichamica,” “*Ca.* M. girerdii” and *M. hominis* had the 4th, 6th and 7th fewest total CDS, respectively (Table 3). However, this did not correspond to a reduced protein family repertoire as “*Ca.* M. trichamica,” “*Ca.* M. girerdii” and *M. hominis* ranked midrange for combined gene families and singletons (17th, 14th and 13th).

**Table 3:** Summary of protein coding genes and gene families among analysed Mollicute genomes.

Genome	Total CDS	Gene families	Singletons	Total*
*Mycoplasma haemofelis*	1,495	323	42	365
*Mycoplasma haemocanis*	1,130	321	24	345
*Mycoplasma testudinis*	1,059	559	205	764
*Mycoplasma penetrans*	1,010	528	150	678
*Mycoplasma iowae*	932	537	174	711
*Mycoplasma suis*	852	310	116	426
*Ureaplasma diversum*	783	420	135	555
*Mycoplasma ovis*	775	309	122	431
*Mycoplasma gallisepticum*	715	485	53	538
*Mycoplasma imitans*	701	487	56	543
*Mycoplasma wenyonii*	696	302	71	373
*Mycoplasma alvi*	678	475	56	531
*Mycoplasma pneumoniae*	676	410	54	464
*Mycoplasma pirum*	667	476	47	523
*Ureaplasma urealyticum*	646	455	49	504
*Ureaplasma canigenitalium*	633	416	80	496
*Ureaplasma parvum*	603	450	35	485
*“Ca.* M. girerdii*”* VCU_M1	572	402	44	446
*“Ca.* M. trichamica”	564	385	46	431
*“Ca.* M. girerdii*”* UC_B3	563	404	33	437
*Metamycoplasma hominis*	557	308	147	455
*Mycoplasma parvum*	544	298	60	358
“*Ca.* Mycoplasma haemominutum”	532	277	70	347
*Mycoplasma genitalium*	503	387	25	412

*Sum of gene families and singletons.

A general feature specific to “*Ca.* M. trichamica” and “*Ca.* M. girerdii” compared with related Mollicutes appeared to be an adaptation towards an anaerobic lifestyle. Related gene families unique to “*Ca.* M. trichamica” and “*Ca.* M. girerdii” within our analysis included Pyruvate:ferredoxin oxidoreductase, rubrerythrin, desulfoferrodoxin, ferrous iron transport protein B and FeoA domain-containing protein (Chabrière et al., 1999; Lombard et al., 2000; Sztukowska et al., 2002; Lau et al., 2016). Additionally, amino acid catabolism was shared feature, indicated by alanine dehydrogenase and L-serine ammonia-lyase gene families also unique to “*Ca.* M. trichamica” and “*Ca.* M. girerdii” within our analysis. Notable other gene families apparently unique to “*Ca.* M. girerdii” and “*Ca.* M. trichamica” included 2′,3′-cyclic-nucleotide 2′-phosphodiesterase, and anaerobic ribonucleoside-triphosphate reductase (ARTR). A single contig (Node 11) assembled from the pigeon metatranscriptomics data shared 96.8% sequence identity at the nucleotide level with the putative ARTR encoded by the “*Ca.* M. trichamica” MAG across its full length. Apparently unique to “*Ca.* M. girerdii” in our analysis was a putative class IIb bacteriocin (Genbank accession: ASJ89026.1).

**Figure 9:**
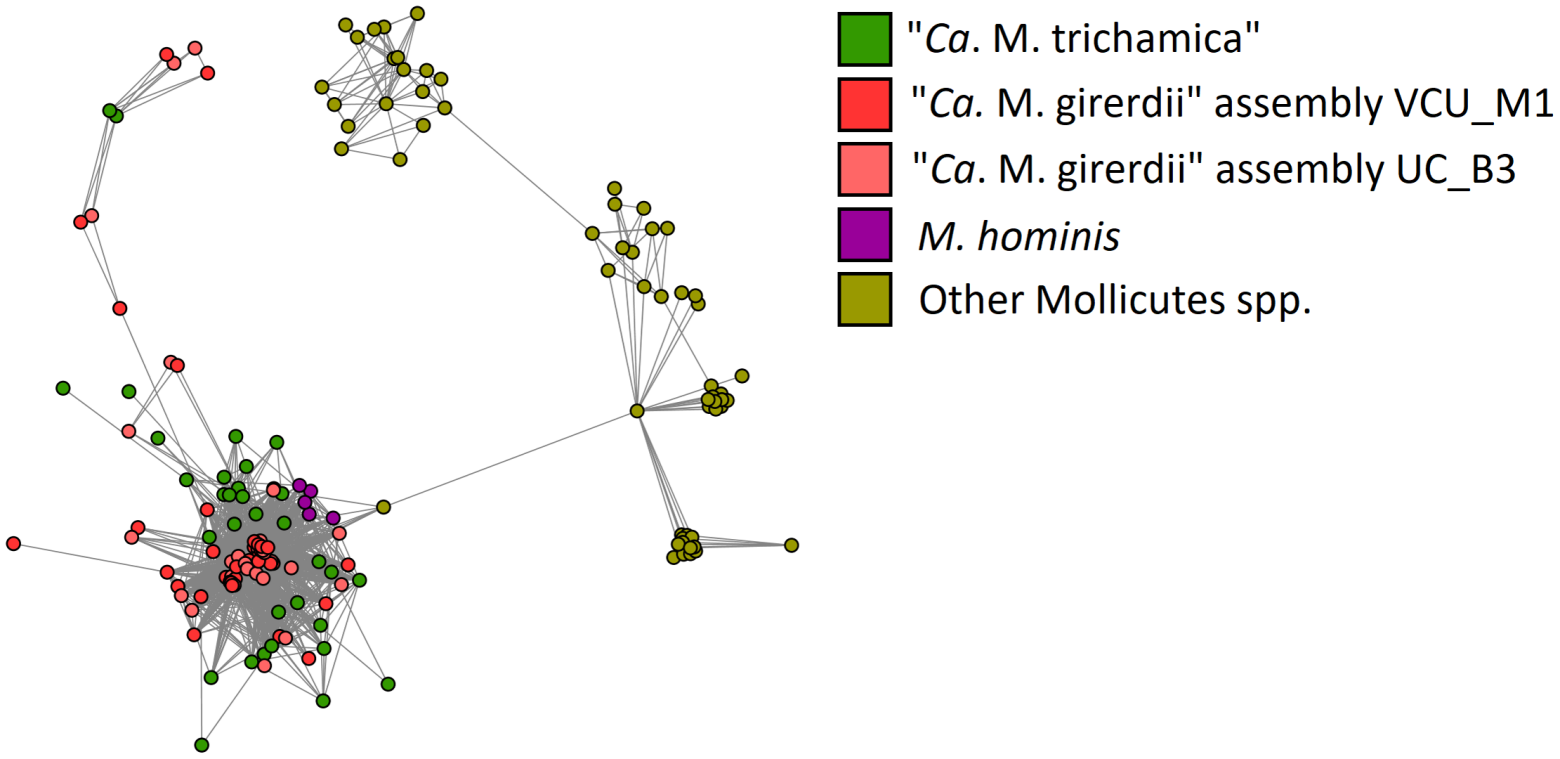
Network representation of the BspA gene family shared among Mollicutes highlighted in Figure 8. Nodes represent CDS which are linked by edges representing BLASTp hits. The layout was determined by the edge-weighted spring-embedded algorithm based on the E-value for BLAST hits (shorter edges represent hits with lower E values). Nodes are coloured according to genome of origin (green; “*Ca.* M. trichamica” MAG generated during this work, red; “*Ca.* M. girerdii” assembly VCU_M1, pink; “*Ca.* M. girerdii” assembly UC_B3, purple; *Mycoplasma hominis* and yellow; all other Mollicutes spp.).

BspAs, a diverse group of cell surface proteins mediating cell–cell interactions (Sharma, 2010), are of interest due to their annotation in the genome of both “*Ca.* M. girerdii” and *T. vaginalis*. In our analysis, BspAs formed a large protein family with 159 members distributed across 11 Mycoplasma spp. genomes. These genomes appear to originate from three separate linages as highlighted in Figure 7. Due to the repetitive nature of BspA proteins (characterised by leucine-rich repeats) phylogenetic analyses are problematic and so we employed gene network analysis to investigate their relationship. Graphical network analysis of the BspA family based on sequence similarity suggested two major distinct groups, one of which was mostly confined to “*Ca.* M. girerdii,” “*Ca.* M. trichamica” and *M. hominis* but also included a single member encoded by the genome of *Mycoplasma pirum* (Figure 9). BspA sequences from “*Ca.* M. girerdii,” *M. hominis* and “*Ca.* M. trichamica” were absent from the other group, which was associated with the first group via a single BLASTp hit (33.9% sequence identity) with *M. pirum*. Clustering of putative *M. hominis* BspAs was apparent from the network, but those from “*Ca.* M. girerdii” and “*Ca.* M. trichamica” were interspersed.

## 4. Discussion

In this work, we identified a close relative of “*Ca.* M. girerdii” in the pigeon upper digestive tract. We propose the name “*Candidatus* Malacoplasma trichamica” to describe this bacterium, justified by its distinct phylogenetic profile and host association. Our results indicated the presence of closely related “*Ca.* M. trichamica” strains in distant geographical locations (China and the United Kingdom) and distinct contexts (farmed and racing pigeons). This suggests that the bacterium is globally distributed among pigeon populations as is known for *T. gallinae* (Amin et al., 2014). However, systematic survey would be required to examine the prevalence of the bacterium among pigeons.

Our findings strongly indicate a close, possibly obligate *in vivo* association between “*Ca.* M. trichamica” and *T. gallinae*, mirroring the strong association observed between *T. vaginalis* and “*Ca.* M. girerdii” (Fettweis et al., 2014; Margarita et al., 2022). Within the crop, “*Ca.* M. trichamica” was found exclusively in *T. gallinae*-infected birds, was among the most strongly associated bacteria with *T. gallinae* in crop and is also possibly co-localised with *T. gallinae* in the crop. Some crop samples showed very high abundance of “*Ca.* M. trichamica,” and similarly high abundances have been observed for “*Ca.* M. girerdii” in the human UGT (Fettweis et al., 2014). Detection of “*Ca.* M. trichamica” in a few gut samples could possibly be explained by transition of material along the digestive system (Stearns et al., 2011). Otu13 sequences were found in the gut of a single bird not infected with *T. gallinae*, which could possibly be explained by a recent clearance of parasitic infection. Interestingly, we did not find a correlation between the abundances of parasite and “*Ca.* M. trichamica.” This could possibly reflect the semi-quantitative approach used to measure parasite abundance by counting parasite cells by wet-mount microscopy (Ji et al., 2020). However, it is also possible that bacterial and parasite abundances are not interdependent, despite their possible unilateral obligate association (with respect to the bacterium). Other factors may support “*Ca.* M. trichamica” growth, such as infection of host cells, as has been observed for “*Ca.* M. girerdii” (Fettweis et al., 2014) and *M. hominis* (Rappelli et al., 2001). Indeed, as was observed for “*Ca.* M. girerdii” (Fettweis et al., 2014), we detected a high abundance of the bacterium is some pigeon crop samples (up to 46% abundance by 16SrRNA profiling). In addition, coverage data from the genome sequence of “*Ca.* M. trichamica” indicated active replication in the pigeon crop, as was suggested for “*Ca.* M. girerdii” in the infant oral cavity (Costello et al., 2017).

Additionally, we reported respective positive and negative correlations between the presence of *T. gallinae* and OTUs assigned as Prevotellaceae and *Lactobacillus*. The latter relationship was previously reported by Ji et al., 2020 based on the same dataset. These findings are of particular interest because Prevotellaceae includes *Prevotella* species which have previously been reported to show a positive association with *T. vaginalis* infection in the human UGT. Similarly, *Lactobacillus* have been negatively correlated with *T. vaginalis* presence in the human UGT (Jarrett et al., 2019; Masha et al., 2019; Chiu et al., 2021). This could indicate a more broadly conserved interaction between members of the *Trichomonas* genus and specific bacterial taxa. We also observed that an OTU closely related to the pigeon respiratory disease-associated bacterium *Pelistega europae* had one of the strongest positive correlations with *T. gallinae* in the crop. We speculate that there could be a connection between the diseases associated with the bacterium (Vandamme et al., 1998) and parasite (Amin et al., 2014). During this work, we generated a genome sequence for “*Ca.* M. trichamica” via metagenomic sequencing. Our initial results suggested that the MAG represents an accurate and complete whole genome sequence. Patterns of sequence composition and read coverage argue against misassembly or sequence chimerism (Chen et al., 2020). Presence of almost the full complement of expected Mollicute single-copy genes further supports genome completeness (Manni et al., 2021). The 54 single copy orthologs encoded by the MAG also provided a congruent phylogenetic signal which was consistent with published Mollicute phylogenies (Fettweis et al., 2014; Gupta et al., 2018) and confirmed a close phylogenetic relationship between “*Ca.* M. girerdii” and “*Ca.* M. trichamica.” This also provides strong evidence against chimerism of the genome. Independent resequencing of a highly similar genome in separate facilities (located in the United Kingdom and China) further supports the validity of the genome sequence.

The “*Ca.* M. trichamica” genome shared substantial similarity and synteny with “*Ca.* M. girerdii,” consistent with their close phylogenetic relationship. A comparison of closely related Mollicutes suggested that reductive evolution appears to be a defining feature of the “*Ca.* M. girerdii”/“*Ca.* M. trichamica” lineage, even in the context of the generally small Mollicute genomes (Razin, 2006).“*Ca.* M. girerdii” and “*Ca.* M. trichamica” had among the fewest total CDS compared with a range of closely related Mollicutes. This reduction could reflect a shift in symbiotic dependence on *Trichomonas* spp. (McCutcheon and Moran, 2011). This is further argued by the reduced genome of *M. hominis* (Calcutt and Foecking, 2015), which is also a symbiont of *Trichomonas* from a separate Mollicute linage (Dessì et al., 2019). Interestingly, “*Ca.* M. girerdii”/“*Ca.* M. trichamica” genome reduction apparently did not coincide with a reduction in protein repertoire, as the number of total protein families was midrange compared with related species. This suggests that the loss of redundant gene functions replaced by the parasite host is not the trigger for observed genome reduction. Transition to an anaerobic glycolytic lifestyle also appears to be a defining feature of “*Ca.* M. girerdii” and “*Ca.* M. trichamica” compared with related species. *Trichomonas* spp. are also considered largely anaerobic (Kulda, 1999), and so this transition may represent an adaptation to symbiosis within the same physiological environment.

Our genomic analysis highlighted several gene families which warrant further investigation into their potential role in “*Ca.* M. girerdii”/“*Ca.* M. trichamica” mucosal survival and symbiosis with *Trichomonas*. We identified a gene putatively encoding 2′,3′-cyclic-nucleotide 2′-phosphodiesterase that was unique to “*Ca.* M. girerdii”/“*Ca.* M. trichamica” compared with related species. We previously showed this gene, which hydrolyses cyclic nucleotides, to be among the most highly expressed genes by “*Ca.* M. girerdii” in symbiosis with *T. vaginalis* (Margarita et al., 2022). In the same system, adenylate/guanylate cyclase genes, which may generate the cyclic nucleotide substrate of the phosphodiesterase, were among the most upregulated by *T. vaginalis* in response to “*Ca.* M. girerdii” presence (Margarita et al., 2022). We speculate that this could underpin a signalling mechanism between the symbiotic parasite and bacteria that could be unique to the “*Ca.* M. girerdii”/“*Ca.* M. trichamica” lineage. Our analysis suggested the gene putatively encoding ARTR is also a feature common to “*Ca.* M. girerdii” and “*Ca.* M. trichamica” and absent in related species. ARTR interconverts ribonucleotide triphosphates and deoxyribonucleotide triphosphates and thus playing a role in DNA synthesis (Eliasson et al., 1990). ARTR was the most highly expressed gene in “*Ca.* M. girerdii” growing in symbiosis with *T. vaginalis* (Margarita et al., 2022), suggesting its functional importance. Similarly, in this work we detected a contig likely to encode “*Ca.* M. trichamica” ARTR via metatranscriptomics analysis of pigeon oral samples. This suggests that the gene is also highly expressed by “*Ca.* M. trichamica” *in vivo*.

BspAs are a diverse family of cell surface proteins implicated in host adhesion and cell–cell adhesion in bacteria (Sharma, 2010). BspAs are of particular interest in *T. vaginalis* biology, as they have a demonstrated influence on adhesion to host cells *in vitro* (Handrich et al., 2019). Importantly for *Trichomonas*-mycoplasma symbiosis, we observed significant upregulation of *T. vaginalis* BspAs response to *Mycoplasma* in a manner dependent on symbiont species (Margarita et al., 2022). Our genomic comparison revealed that the *Trichomonas*-symbiotic “*Ca.* M. girerdii,” “*Ca.* M. trichamica” and *M. hominis* share a family of BspA proteins. The distribution of BspAs among “*Ca.* M. girerdii,” “*Ca.* M. trichamica,” *M. hominis* and related species can be explained with roughly equal parsimony by differential acquisition by three linages or loss by two lineages. However, the relatively strong sequence similarity between “*Ca.* M. girerdii,” “*Ca.* M. trichamica” and *M. hominis* BspAs (range 22–100%, mean 42%), and more distant similarity to BspAs in the *Mycoplasma gallisepticum* lineage suggests their independent acquisition. Integrating this evidence, we hypothesise that *T. vaginalis* and *Mycoplasma* BspAs may play an important role during symbiosis, such as in cell–cell recognition between the eukaryote and the bacteria. The clustering pattern of *M. hominis* BspAs among the “*Ca.* M. trichamica” BspAs in the gene network analysis and the distinct phylogenetic position of *M. hominis* relative to the “*Ca.* M. girerdii” /“*Ca.* M. trichamica” lineage among Mollicutes also suggests a potential lateral gene transfer (LGT) between the Mycoplasma species for the BspA encoding genes.

**Figure 10:**
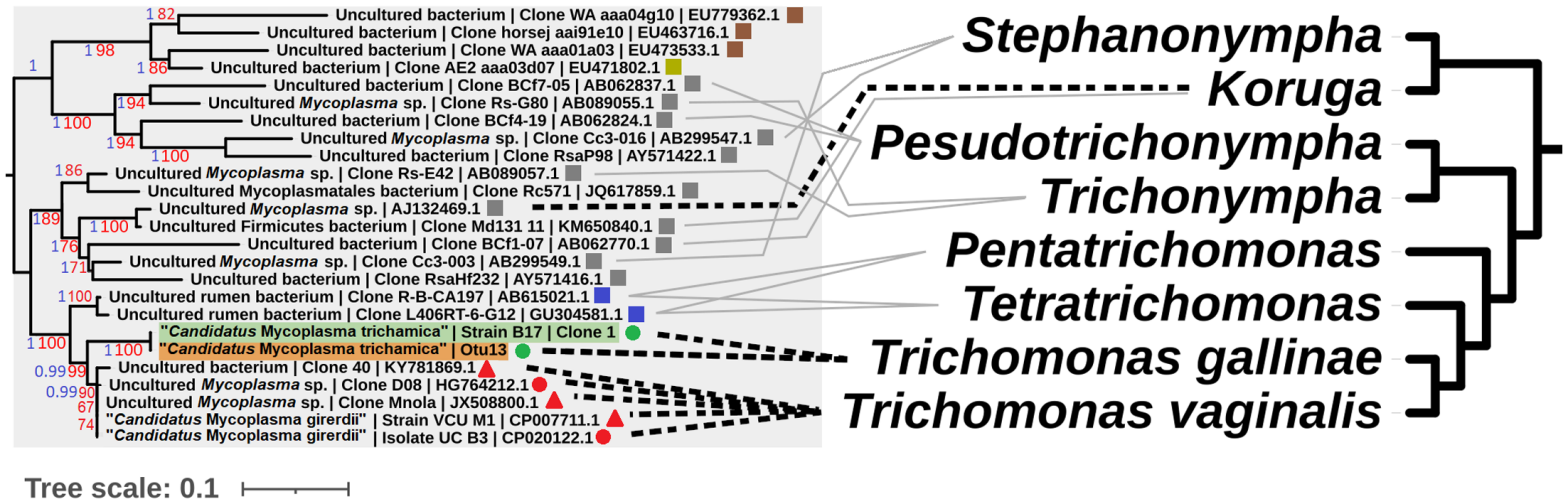
Illustrative cophylogeny of Parabasalia and Mollicutes species. The left phylogeny is derived from the Mollicutes phylogeny in Figure 2, including only the lineage containing “*Ca.* M. girerdii” and related bacteria. Coloured shapes indicate the isolation source for the bacteria (red; human, green; pigeon, blue; cattle, grey; termite, brown; equine, yellow; elephant, round; upper GI, square; lower GI, triangle; urogenital). Pigeon-derived sequences from this study and 16S profiling by (Ji et al., 2020) are highlighted in green and orange, respectively. Bootstrap support values (1,000 replicates) greater than 65% and aBayes values greater than 0.95 are shown on branches in red and blue, respectively. Where available, GenBank accessions for each sequence are shown at the ends of tip labels. The right cladogram has been adapted from the phylogenetic relationships within Parabasalia described by Noda et al. (2012), with the position of Koruga inferred from Cepicka et al. (2010). Black dashed lines connect taxa for which either a physical association or statistical association of occurrence has been demonstrated *in vivo* (Fröhlich and König, 1999; Costello et al., 2013; Martin et al., 2013; Fettweis et al., 2014; Costello et al., 2017; Ioannidis et al., 2017). Grey lines indicate taxa which have been detected within the same host species and mucosal site for cattle (Jensen and Hammond, 1964; Calcutt and Foecking, 2015; Zhang et al., 2020) and termites (Noda et al., 2012).

A gene feature apparently unique to “*Ca.* M. girerdii” and absent in “*Ca.* M. trichamica” and other close relatives encodes a putative class IIb bacteriocin. Bacteriocins are a class of antimicrobial peptides which mediate interspecific competition between bacteria. Thus, this putative bacteriocin in “*Ca.* M. girerdii” could mediate competition with other microbes in the human UGT. “*Ca.* M. girerdii” and *M. hominis* naturally co-infect *T. vaginalis* strains at a high prevalence (Xu et al., 2021; Margarita et al., 2022). Intriguingly, previous work has suggested a potentially competitive interaction between “*Ca.* M. girerdii” and *M. hominis* abundance, which could be explained by interspecific competition via mechanisms including bacteriocins (Margarita et al., 2022). We propose that “*Ca.* M. girerdii” emerged during a cross-species transmission from birds to humans, alongside the bird-infecting ancestor of *T. vaginalis*, possibly harboured within the cells of the parasite. As the relationship between the bacteria and parasite appears to have been evolutionarily conserved, it could be speculated that the association may be more deeply rooted within Parabasalia. There is an interesting parallel between the phylogenies of Parabasalids and uncultured Mollicute species more distantly related to “*Ca.* M. girerdii,” many of which have been isolated from the same host species (Figure 10). Remarkably, an uncultured Mollicute within the putative Parabasalia-associated linage (GenBank accession AJ132469.1) was isolated directly from the cytoplasm of the termite-associated parabasalid *Koruga bonita* by micromanipulation (Fröhlich and König, 1999). Future work should search for associations between Mollicutes and a greater range of Parabasalia species.

Interestingly, we found no evidence for close relatives of *M. hominis* in the pigeon system. The 16S rRNA sequences of Mollicutes within the *M. hominis* cluster have been shown to share 94% similarity (Pettersson et al., 2000), and so are likely to have been readily identifiable by this study. However, sampling of a greater number of birds over a wide geographic distribution would be necessary to further support the absence of *M. hominis* relatives. The closest relatives of *M. hominis* infect a range of predominantly mammalian hosts, with the horse-associated *Mycoplasma equirhinis* indicated as the most closely related (Pettersson et al., 2000). In addition, the capacity of *M. hominis* to cause UGT infection independently of *T. vaginalis* presence may suggest its pre-existence in the human UGT. Thus, *M. hominis* may not share an avian origin with “*Ca.* M. girerdii,” and symbiosis with *T. vaginalis* may have arisen independently.

In summary, we report an association between *T. gallinae* and a previously undescribed Mollicutes species in the pigeon upper digestive tract. This, and other data (Fröhlich and König, 1999) support a potential long evolutionary history of association between Parabasalia and a specific lineage of Mollicutes. This demonstrates the potential evolutionary stability of symbiotic relationships between mucosal pathogens and other members of the microbiota. Our genomic analysis suggested features specific to the *Trichomonas*-associated Mollicute lineage which could provide a functional basis for the symbiosis, which should be the basis for further investigation. The impact of symbiotic relationships with other microorganisms should be considered in order to more fully understand the behaviour of important human pathogens such as *T. vaginalis* (Rowley et al., 2019).

## Data availability statement

All the sequencing data and corresponding assemblies can be accessed from the NCBI with the following accessions: BioProject PRJNA797924: Metagenome, China, SRA: SRR17641486. Assembled genome, China, biosample: SAMN25041167. Metagenome, UK, SRA: SRR18744026. Metatranscriptome, UK, SRA: SRR17643316 AND SRR17643315. BioProject PRJNA816903: assembled genome, UK: assembly accession: SAMN25134305. Additional data are available in the supplementary material.

## Ethics statement

Ethical approval was not required for the studies involving animals in accordance with the local legislation and institutional requirements because the oral cavity of pigeons was swabbed while manipulated by the owner. This was performed following specific training required to obtain the specific licence from “Nature England” allowing manipulation of birds, including swabbing. Written informed consent was obtained from the owners for the participation of their animals in this study.

## Author contributions

NB, RH, and NH conceived and designed the analysis. NB, YS, and SD collected the data. NB, PF, JF, and RH performed the analysis. NB, RH, and PF wrote the manuscript. ZW contributed to training and supervision of YS and SD. RH and ZW were responsible for project management and funding access. All authors contributed to the article and approved the submitted version.

## Funding

This work was supported by the Biotechnology and Bioscience Research Council Doctoral Training Partnership for Newcastle, Liverpool and Durham (grant number: BB/M011186/1 – NPB PhD student, RH supervisor). Work in China was funded by Innovation capacity building project of Beijing Academy of Agriculture and Forestry Science (KJCX 20200404), and Beijing municipal science and technology commission national modern agricultural science and technology city industry cultivation and achievement benefit project (Z171100001517003). NH is supported by the Core strategic Program of the Earlham Institute BB/CCG1720/1. Next-generation sequencing of the UK derived sample was delivered via the BBSRC National Capability in Genomics and Single Cell Analysis BB/CCG1720/1 at Earlham Institute.

## Conflict of interest

The authors declare that the research was conducted in the absence of any commercial or financial relationships that could be construed as a potential conflict of interest.

## Publisher’s note

All claims expressed in this article are solely those of the authors and do not necessarily represent those of their affiliated organizations, or those of the publisher, the editors and the reviewers. Any product that may be evaluated in this article, or claim that may be made by its manufacturer, is not guaranteed or endorsed by the publisher.
