# Supplementary material for: Evolutionary conservation of Trichomonas-mycoplasma symbiosis across the host species barrier
Source: Front Microbiol. 2023 Sep 20;14:1242275. doi: 10.3389/fmicb.2023.1242275 (PMC10557491; doi:10.3389/fmicb.2023.1242275)
Supplement: Supplementary file 5 [file Data_Sheet_3.pdf]

Supplementary Material

Evolutionary conservation of Trichomonas-Mycoplasma symbiosis across the host species barrier

Nicholas P. Bailey[1*†], Yuxin Shao[2†], Shaodua Du[2], Peter G. Foster[3], Jennifer Fettweis[4], Neil Hall[5,6], Zheng Wang[2*], Robert P. Hirt[1*]

*** Correspondence:** Corresponding Authors
n.bailey2@newcastle.ac.uk, wz7324@163.com and Robert.Hirt@newcastle.ac.uk

1 Supplementary Data

Supplementary Data include two files:

Supplementary_Data_File_1_RNASeq_contigs.fasta

Supplementary_Data_File_2_concat83_13845.fasta

2 Supplementary Figures and Tables

There are two Supplementary tables:

Supplementary_Table_1.xlsx

Supplementary_Table_2.xlsx

2.1 Supplementary Figure

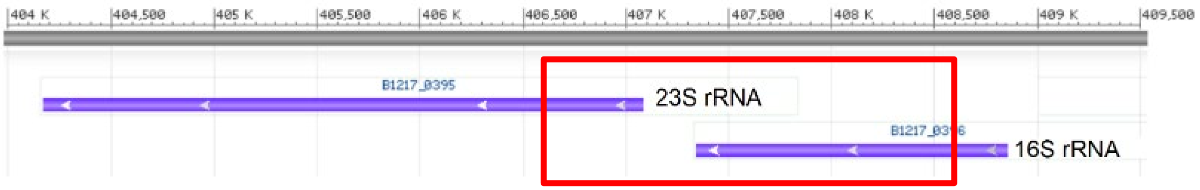

Supplementary Figure 1. Schematic representation of the rRNA locus from "Ca. *M. girerdii*". Red square indicates the region targeted for PCR by the generic bacterial 16S rRNA forward primer 338F and "Ca. *M. trichamica*"-specific 23S rRNA reverse primer. Adapted from the NCBI sequence viewer (Sayers, et al. 2019)..

Reference

Sayers EW, Agarwala R, Bolton EE, Brister JR, Canese K, Clark K, Connor R, Fiorini N, Funk K, Hefferon T, et al. 2019. Database resources of the National Center for Biotechnology Information. Nucleic Acids Res 47:D23-D28.
